# Paramedics assessing patients with complex comorbidities in community settings: results from the CARPE study

**DOI:** 10.1007/s43678-021-00153-4

**Published:** 2021-08-17

**Authors:** Matthew S. Leyenaar, Brent McLeod, Aaron Jones, Audrey-Anne Brousseau, Eric Mercier, Ryan P. Strum, Michael Nolan, Samir K. Sinha, Gina Agarwal, Walter Tavares, Andrew P. Costa

**Affiliations:** 1 Prince Edward Island Department of Health and Wellness, Charlottetown, PEI Canada; 2 Department of Health Research Methods, Evidence, and Impact, McMaster University, Hamilton, ON Canada; 3 Hamilton Niagara Haldimand Brant Local Health Integration Network, Grimsby, ON Canada; 4 Département de Médecine d’urgence, Université de Sherbrooke, Sherbrooke, QC Canada; 5 Département de Médecine Familiale et Médecine d’Urgence, Faculté de Médecine de l’Université Laval, Quebec, QC Canada; 6 County of Renfrew Paramedic Service, Pembroke, ON Canada; 7 Sinai Health, Toronto, ON Canada; 8 Faculty of Medicine, Institute of Health Policy, Management and Evaluation, Dalla Lana School of Public Heath, University of Toronto, Toronto, ON Canada; 9 York Region Paramedic Services, Regional Municipality of York, Sharon, ON Canada; 10 Department of Family Medicine, McMaster University, Hamilton, ON Canada

**Keywords:** Emergency medical services, Community paramedicine, Case finding, Standardized assessment

## Abstract

**Objectives:**

The aim for this study was to provide information about how community paramedicine home visit programs best “navigate” their role delivering preventative care to frequent 9-1-1 users by describing demographic and clinical characteristics of their patients and comparing them to existing community care populations.

**Methods:**

Our study used secondary data from standardized assessment instruments used in the delivery of home care, community support services, and community paramedicine home visit programs in Ontario. Identical assessment items from each instrument enabled comparisons of demographic, clinical, and social characteristics of community-dwelling older adults using descriptive statistics and *z*-tests.

**Results:**

Data were analyzed for 29,938 home care clients, 13,782 community support services clients, and 136 community paramedicine patients. Differences were observed in proportions of individuals living alone between community paramedicine patients versus home care clients and community support clients (47.8%, 33.8%, and 59.9% respectively). We found higher proportions of community paramedicine patients with multiple chronic disease (87%, compared to 63% and 42%) and mental health-related conditions (43.4%, compared to 26.2% and 18.8% for depression, as an example).

**Conclusion:**

When using existing community care populations as a reference group, it appears that patients seen in community paramedicine home visit programs are a distinct sub-group of the community-dwelling older adult population with more complex comorbidities, possibly exacerbated by mental illness and social isolation from living alone. Community paramedicine programs may serve as a sentinel support opportunity for patients whose health conditions are not being addressed through timely access to other existing care providers.

**Protocol registration:**

ISRCTN 58273216.

**Supplementary Information:**

The online version contains supplementary material available at 10.1007/s43678-021-00153-4.

## Résumé

**Objectifs:**

L'objectif de cette étude était de fournir des informations sur la façon dont les programmes de visites à domicile des paramédicaux communautaires " naviguent " le mieux possible dans leur rôle de prestation de soins préventifs aux utilisateurs fréquents du 9-1-1 en décrivant les caractéristiques démographiques et cliniques de leurs patients et en les comparant aux populations de soins communautaires existantes.

**Méthodes:**

Notre étude a utilisé des données secondaires provenant d’instruments d’évaluation normalisés utilisés dans la prestation de soins à domicile, de services de soutien communautaire et de programmes de visites à domicile paramédicaux communautaires en Ontario. Des éléments d’évaluation identiques de chaque instrument ont permis de comparer les caractéristiques démographiques, cliniques et sociales des personnes âgées vivant dans la collectivité à l’aide de statistiques descriptives et de tests z.

**Résultats:**

Les données ont été analysées pour 29 938 clients des soins à domicile, 13 782 clients des services de soutien communautaire et 136 patients des services paramédicaux communautaires. Des différences ont été observées dans les proportions de personnes vivant seules entre les patients paramédicaux communautaires par rapport aux clients des soins à domicile et aux clients du soutien communautaire (47,8%, 33,8% et 59,9% respectivement). Nous avons trouvé des proportions plus élevées de patients paramédicaux communautaires atteints de maladies chroniques multiples (87%, contre 63% et 42%) et de problèmes de santé mentale (43,4%, contre 26,2% et 18,8% pour la dépression, par exemple).

**Conclusion:**

En utilisant les populations de soins communautaires existantes comme groupe de référence, il semble que les patients vus dans les programmes de visites à domicile paramédicaux communautaires soient un sous-groupe distinct de la population des personnes âgées vivant dans la collectivité avec des comorbidités plus complexes, peut-être exacerbées par la maladie mentale et l’isolement dû au fait de vivre seul. Les programmes paramédicaux communautaires peuvent servir de soutien sentinelle pour les patients dont l'état de santé n'est pas pris en charge par le biais d'un accès rapide à d'autres prestataires de soins existants.

**Clinician’s capsule**

***What is known about the topic?***

Community paramedicine programs are designed to improve access to care for vulnerable patient groups but case finding remains a challenge.

***What did this study ask?***

We investigated who community paramedicine patients are and how they compare to clients receiving community care using identical assessment items.

***What did this study find?***

Community paramedicine patients have higher proportions of multiple chronic disease and mental health-related conditions than others who receive community care.

***Why does this study matter to clinicians?***

Community paramedicine programs support older patients with complex comorbidities and mental illness by offering improved access to collaborative care.

## Introduction

Community paramedicine programs address barriers to care faced by community-dwelling older adults (≥ 65 years of age) or other vulnerable patient populations who may otherwise resort to calling an ambulance or visiting an emergency department (ED) [1–4]. Community paramedicine home visit programs have improved access to care for frequent callers through collaboration between primary care providers and community home care and support services agencies [4–9] resulting in patients avoiding ED visits upwards of 78% of the time and higher admission rates when visits are unavoidable [10]. Frequent callers use paramedic services for reasons beyond acute medical emergencies including to address personal or social care needs (such as loneliness, food insecurity, or other deficits in quality of life), chronic conditions (such as pain, disease, or ongoing management of mental health), or functional and mobility difficulties related to advanced age [11–16]. Across Canada, expansion of community paramedicine from pilot projects to province-wide programs [17–20] has been supported by a growing evidence base [5–7, 9, 21–24]. Community paramedicine programs are attempting to shift from “reactive responses” towards better management of chronic conditions with fewer exacerbations [1] by targeting frequent callers who represent up to 20% of ED visits [12].

Whether community paramedicine home visit programs represent a duplication of community-based services requires further exploration [14, 25, 26]. Studies have found that home care nursing visits are associated with same-day ED visits [27], that home care clients use paramedic services for transportation to the ED for such visits [16], and that paramedic referrals are associated with increased utilization of home care services [28]. If home care clients present with lower acuity levels at an ED visit and are not admitted to hospital [27], more information is needed to determine how community paramedics could better “navigate” their role in the delivery of integrated care [25, 26]. While community paramedicine home visit programs often incorporate collaborations with other out-of-hospital community care programs [5], an expanded description about the demographic and clinical characteristics of community paramedicine patients that includes comparisons with existing community care populations would demonstrate the role community paramedicine programs play in case-finding individuals for the delivery of out-of-hospital community care and prevention of ED visits. Implementation of the PERIL rule [29] to inform paramedic referrals to home care services demonstrated how paramedic screening at the time of a 9-1-1 call increased appropriate provision of home care services [28]. Assessments by community paramedics capturing a wide breadth of clinical observations could demonstrate similar utility in community paramedicine home visit programs, even where patient enrollment is determined by local program design [30], and guide further coordination between primary care providers, home care, and community support services [31].

Our study proposed to identify characteristics of existing community paramedicine home visit patients across multiple jurisdictions and compare them to clients from other community-based care providers. We hypothesized that patients in community paramedicine home visit programs represent a distinct subset of community-dwelling older adults with complex needs and a limited social support structure that contributes to their enrolment in these programs.

## Methods

### Overview

Our study used routinely collected de-identified secondary data about individuals assessed for their eligibility of home care services or as part of the delivery of community support services or community paramedicine programs across Ontario. We used identical variables from each data set to compare the home care and community support services client populations to those enrolled in community paramedicine home visit programs. This study was approved by Hamilton Integrated Research Ethics Board (#1650D).

### Study settings and population

#### Home care clients

Information about home care clients included all individuals assessed using the interRAI Home Care (HC) assessment [32], between April 1, 2018 and March 31, 2019 in one health region in Ontario, Canada. The interRAI-HC is a mandatory standardized assessment instrument for individuals that are expected to receive home care services for 60-days or more [33]. The Canadian Institute for Health Information’s Home Care Reporting System is a repository of interRAI-HC data used for epidemiologic research and reporting on quality measures [33].

#### Community support services clients

Information about clients receiving community support services was obtained from individuals who had been assessed using the interRAI Community Health Assessment (CHA) assessment instrument [34], between January 1, 2017 and December 31, 2017 (the most recent year for which data were available) in multiple jurisdictions across Ontario. The interRAI-CHA includes the same assessment domains as the interRAI-HC but uses a modular design [34]. For example, assessors could be alerted to the need for a more detailed mental health assessment for some clients thereby completing these assessment items only on those where it was indicated and not others. The interRAI-CHA is used by community support services to assess individuals who receive services like homemaking, friendly-visiting, or adult day programs [33]. Agencies establish their own parameters for use of the interRAI-CHA and share data with the interRAI Canada Repository [35]. Both the interRAI-HC and interRAI-CHA include decision support scales and screeners that have undergone extensive testing with demonstrated validity and reliability [33, 36].

#### Community paramedicine home visit program patients

Data about individuals enrolled in community paramedicine home visit programs were obtained from paramedic services that implemented a standardized assessment instrument as part of the Common Assessments for Repeated Paramedic Encounters (CARPE) study (ISRCTN 58273216). Several paramedic services participated in development of the CARPE assessment instrument through a process including literature review [5], expert panel consultation [30], and an environmental scan of community paramedicine assessment practices [37].

Six paramedic services implemented the CARPE assessment instrument voluntarily as part of a quality improvement process within existing community paramedicine home visit programs. All paramedic services had similar patient enrollment criteria: diagnoses of Congestive Heart Failure (CHF), Chronic Obstructive Pulmonary Disease (COPD), or diabetes, and health system utilization that included at least three 9-1-1 calls, two ED visits, or one hospital admission in the preceding year [17, 38]. Community paramedics participated in a 4-hour training session about assessment practices (delivered by a member of the research team in collaboration with local paramedic service educators), received an assessment instrument training manual, and could attend teleconference sessions to clarify any remaining questions. The CARPE assessment instrument was embedded in electronic medical record software and data were provided to the research team for the period from April 1, 2018 to March 31, 2019. The CARPE assessment instrument was used to assess any patient enrolled in a community paramedicine home visit program (herein called community paramedicine patients), either during their enrolment or as part of regular reassessments.

### Analysis

Data were analyzed using descriptive statistics (including calculation of standard error) for each identical item from the respective assessment instruments (see supplemental file Table S1 for variable list). For reporting purposes, items were grouped by domain and collapsed into dichotomous variables to identify presence of disease, health deficits, or indicators of impairment (according to the nature of the respective assessment item). Comparative analysis for each assessment item tested proportions of responses using z-test (with ∝  = 0.05) to investigate differences between the community paramedicine patients and the other cohorts of community-dwelling older adults according to identical fields from the respective assessment instruments. Analysis was completed using SAS 9.4 (SAS Institute Inc, Cary, NC) and excluded incomplete or partial assessments.

## Results

Table 1 provides the demographic characteristics, living conditions, and health system utilization data for each group; 29,938 individuals assessed with the interRAI HC, 13,782 individuals assessed with interRAI CHA, and 136 individuals assessed with the CARPE assessment instrument. Mean ages were 78.8 (SD ± 13.5), 78.2 (SD ± 13.7), and 75.7 (SD ± 14.2) for home care clients, community support services clients, and community paramedicine patients respectively. The proportions of female patients—60.3%, 68.2%, and 64.0%—suggested scant evidence of differences in gender representation between groups. Differences in proportions of individuals living alone was evident, with more community support clients (59.9%) and fewer home care clients (33.8%) when compared to community paramedicine patients (47.8%). The proportion of patients admitted to hospital in the past 90 days was not significantly different when comparing community paramedicine patients to home care clients—47.1% and 41.9% respectively—higher than the proportion observed in community support services clients, 13.6%.

**Table 1:** Demographic and health system utilization of community paramedicine home visit patients, home care clients, and community support agency clients

	Home care* N* = 28,938%	Community support services * N* = 13,782%	Community paramedicine * N* = 136%
Demographic characteristics and living conditions			
Age*	78.6	78.8	75.7
Gender female	60.3	68.2	64.0
Lives alone	***33.8***	***59.9***	***47.8***
Home in disrepair	***4.0***	–	***17.6***
Squalid conditions	***2.4***	–	***14.0***
Inadequate heating or cooling	***0.9***	–	***16.2***
Lack of personal safety	***1.6***	–	***11.0***
Limited access to home or rooms	***17.8***	–	***25.0***
Health system utilization			
Hospital admission in past 90 days	41.9	***13.6***	***47.1***
Called 9-1-1 past 90 days	–	–	53.7
Called 9-1-1 past 30 days	–	–	33.8
At high risk for future ED visit**	***25.0***	–	***15.0***

*Bold* *italics* indicate evidence of statistically significant differences between proportions in comparator groups against community paramedicine patients using *z*-test at α = 0.05

*Data are reported as mean and standard deviation

**Determined from the Detection of Indicators and Vulnerabilities for Emergency Room Trips (DIVERT) Scale, values greater than or equal to 5. The DIVERT scale is used to identify risk for an unplanned emergency department visit in the 90 days following assessment

### Clinical characteristics, chronic disease diagnoses and health conditions

**Fig. 1:**
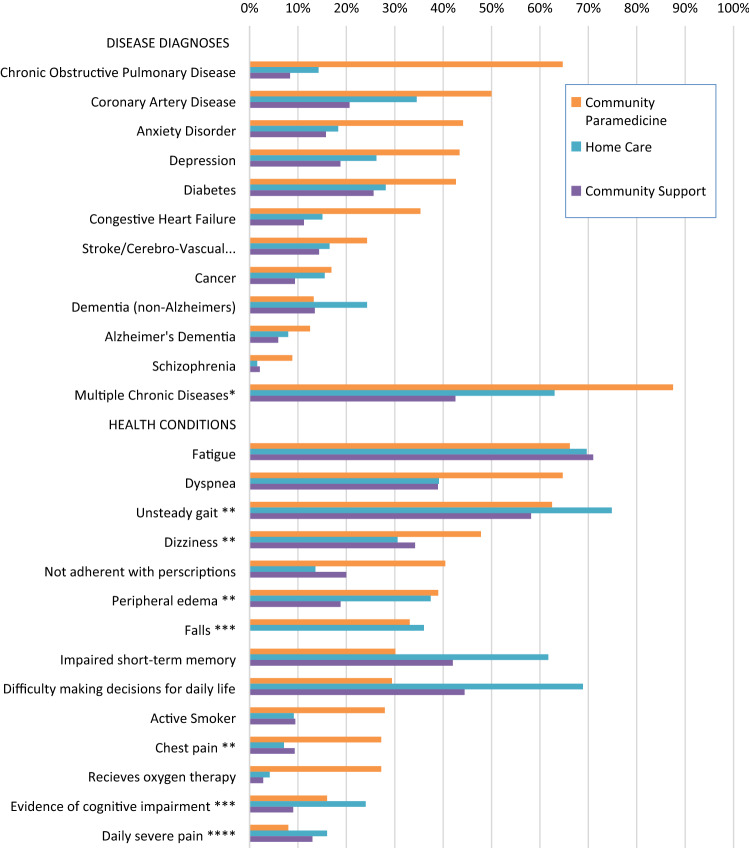
Percentages of comorbidities and multiple health conditions of Community Paramedicine home visit patients, Home Care clients, and Community Support Services Agency clients. Patients may have more than one condition. *Multiple chronic diseases indicates patients with more than one of the diseases diagnoses listed. **Health conditions occurring over 3 days preceding assessment. ***Evidence of cognitive impairment as determined by a score greater than or equal to 2 on the Cognitive Performance Scale. ****Daily severe pain as determined by a combination of responses regarding pain experienced over preceding 3 days

Community paramedicine patients demonstrated higher proportions of COPD, coronary artery disease, diabetes, or CHF (64.7%, 50.0%, 42.6%, and 35.3% respectively compared to 14.3%, 34.5%, 28.1%, and 15.0% in home care clients and 8.4%, 20.7%, 25.6%, and 11.2% in community support services clients, see Fig. 1). They experienced more episodes of dyspnea, dizziness, or chest pain (64.7%, 47.8%, and 27.2% respectively compared to 39.1%, 30.6%, and 7.1% in home care clients and 38.9%, 34.2%, and 9.3% in community support services clients). A higher proportion of community paramedicine patients had multiple chronic diseases (87.5% compared to 63.0% in home care clients and 42.5% in community support services clients). There was a lower proportion of community paramedicine patients who were non-smokers (72.1% compared to 90.9% in home care clients and 90.5% in community support services clients) and a higher proportion who were not adherent with their prescription medications (40.4% compared to 13.6% in home care clients). Statistically significant differences between the community paramedicine patients and the other groups were observed in all of these comparisons.

### Mental health related conditions and psycho-social well-being

Higher proportions of anxiety disorder and depression were found in community paramedicine patients (44.1 and 43.4% respectively compared to 18.3%, and 26.2% in home care clients and 15.8% and 18.8% in community services support clients) and they displayed higher proportions of associated symptoms (See Fig. 2, included in Appendix)—all of which were statistically significant differences. A higher proportion of community paramedicine patients would meet the criteria for further assessment of their mental health, 29.4% when compared to 12.4% in community support services clients. More community paramedicine patients indicated that they had experienced a major life stressor or a decline in social activities in the past 90 days (52.2% compared to 22.5% in home care clients and 25.7% in community support services clients).

### Communication and functional abilities

When comparing community paramedicine patients to community support services clients, little evidence of difference was observed in the proportions of individuals who had difficulty communicating or functional deficits for some Activities of Daily Living and Instrumental Activities of Daily Living (specifically personal hygiene, dressing lower body, transportation, and phone use) (see Fig. 3, included in Appendix). But the home care client group displayed statistically significant differences, with higher proportions of dependence for all functional items when compared to community paramedicine patients.

## Discussion

We found that patients in community paramedicine home visit programs likely represent a distinct sub-group of the community-dwelling older adult population because of numerous differences observed between our cohort groups. Our data suggests that the proportion of individuals with mental health needs, complex co-morbidities, and ongoing health conditions or symptoms are often different between community paramedicine programs and home care and community support services agency populations. Higher proportions of health needs in community paramedicine patients suggests they are a complex patient group who could benefit from more integrated care that includes an interface between multiple care providers—reinforcing a characteristic of many community paramedicine home visit programs [5, 17]. By illustrating differences between community paramedicine home visit patients and other community-dwelling older adults, efforts can support case-finding by all care providers to improve patient care access and reduce unnecessary utilization of 9-1-1 or EDs by these individuals.

### Strengths and weaknesses of the study

Duplication of services with other existing community-based health care services is a critique of community paramedicine programs [25] but, to our knowledge, our study is the first to compare the characteristics of the older adults that are receiving these services. While community paramedicine programs looking to serve frail older adults may have targeted enrollment efforts favouring these individuals, the differences we found suggests duplication of services is unlikely because complex comorbidities, likely exacerbated by mental illness, appeared less common amongst individuals receiving community support services or home care programs. Even if other programs or services are providing care to such patients, community paramedicine programs are likely providing a necessary additional level of support to the existing supports patients may be receiving from community-based care providers. For example, remote patient monitoring programs (broadly implemented in Ontario) support chronic disease management by identifying patients’ worsening health symptoms prior to exacerbations that require a 9-1-1 response while complementing existing care from other community-based care providers [17, 38].

The inferences drawn from our comparisons should be made cautiously because they are based on a small convenience sample of community paramedicine patients assessed using a prototype assessment instrument. Community paramedicine programs remain relatively small in comparison to the number of clients seen through other established programs delivering community-based care. While the sizes of the sample cohorts present a limitation to the inferences, they are reflective of the differences in sizes of the patient populations and were large enough to power statistical analyses. To further strengthen our analysis, we excluded small counts (< 10) of observations from the community paramedicine cohort.

### Implications for clinicians and health policy

Opportunity exists for further collaboration between community-based support services agencies and home care providers, community paramedicine home visit programs, and other parts of the healthcare continuum—particularly primary care providers—to improve coordination of care to medically complex community-dwelling older adults [3, 10, 39]. For example, a risk scale used to determine the likelihood of an ED visit in home care clients is a likely predictor for use of paramedic services for transportation to the ED [16]. Shared case-finding to identify at-risk patients could support greater coordination between hospitals, home care providers, community support services agencies and community paramedicine programs and lead to improved patient safety and reduce unnecessary ED visits and 9-1-1 utilization.

### Implications for future research

Anonymized data were obtained for our study meaning that analysis of cross-membership between cohorts was not possible. It is possible that a handful of patients could have been represented in all three groups and questions remain about the likelihood of this. High levels of cross-membership between groups would have lessened the likelihood of observing differences in our analysis. Future research should aim to provide a complete analysis of health system utilization amongst community dwelling older adults.

High proportions of mental health-related conditions were identified in community paramedicine patients. Other research has demonstrated that mental health and social isolation can contribute to repeated 9-1-1 use [11, 14, 15, 40]. While we provided a comparison to other cohorts of community-dwelling older adults, further comparisons are needed with additional community and geriatric mental health populations. Community paramedicine programs should explore further integration with local community support services agencies and home care providers as part of their program design and community paramedics may benefit from greater education about addressing mental health needs, particularly amongst older adults.

## Conclusion

Our analysis showed that community-dwelling older adults in community paramedicine home visit programs may represent a distinct patient group with a greater proportion of mood symptoms, ongoing health conditions, and complex comorbidities than comparable patient populations that receive home care or community support services. Enrolment into a community paramedicine home visit program may be indicative of a combination of inadequate social support structures or clinical instability and decline of a patient’s condition. Community paramedicine home visit programs may provide a sentinel support opportunity for community-dwelling older patients whose health conditions are not otherwise being addressed through timely access to other existing care providers.

## Supplementary Information

Below is the link to the electronic supplementary material.

Supplementary file1 (DOCX 16 kb)

Supplementary file2 (DOCX 24 kb)

## References

[1] Cameron P, Carter A (2019). Community paramedicine : a patch, or a real system improvement ?. CJEM Can J Emerg Med Care.

[2] Martin A, O’Meara P, Farmer J (2016). Consumer perspectives of a community paramedicine program in rural Ontario. Aust J Rural Health.

[3] Iezzoni LI, Dorner SC, Ajayi T. Community paramedicine—addressing questions as programs expand. N Engl J Med [Internet]. 2016;374(12):1107–9. Available from: http://www.nejm.org/. 10.1056/NEJMp1516100 27007957

[4] Choi BY, Blumberg C, Williams K (2016). Mobile integrated health care and community paramedicine : an emerging emergency medical services concept. Ann Emerg Med [Internet].

[5] Leyenaar M, Mcleod B, Chan J, Tavares W, Costa A, Agarwal G (2018). A scoping study and qualitative assessment of care planning and case management in community paramedicine. Ir J Paramed.

[6] Chan J, Griffith LE, Costa AP, Leyenaar MS, Agarwal G (2019). Community paramedicine: a systematic review of program descriptions and training. CJEM [Internet].

[7] Rasku T, Kaunonen M, Thyer E, Paavilainen E, Joronen K (2019). The core components of community paramedicine—integrated care in primary care setting: a scoping review. Scand J Caring Sci [Internet].

[8] Patterson DG, Coulthard C, Garberson LA, Wingrove G, Larson EH. What is the potential of community paramedicine to fill rural health care gaps? J Health Care Poor Underserved [Internet]. 2016; 27(4A):144–58. Available from: https://muse.jhu.edu/article/634884 10.1353/hpu.2016.0192 27818420

[9] O’Meara P (2014). Community paramedics : a scoping review of their emergence and potential impact. Int Paramed Pract.

[10] Abrashkin KA, Washko J, Zhang J, Poku A, Kim H, Smith KL (2016). Providing acute care at home : community paramedics enhance an advanced illness management program—preliminary data. J Am Geriatr Soc [Internet].

[11] Edwards MJ, Bassett G, Sinden L, Fothergill RT. Frequent callers to the ambulance service: patient profiling and impact of case management on patient utilisation of the ambulance service. Emerg Med J [Internet]. 2014; 32(5):392–6. Available from: http://www.ncbi.nlm.nih.gov/pubmed/25312857 10.1136/emermed-2013-203496 25312857

[12] Scott J, Strickland AP, Warner K, Dawson P. Frequent callers to and users of emergency medical systems: a systematic review. Emerg Med J [Internet]. 2014; 31(8):684–91. Available from: http://www.ncbi.nlm.nih.gov/pubmed/23825060 10.1136/emermed-2013-202545 23825060

[13] Mahmuda S, Wade-Vallance A, Stosic A, Guenter D, Howard M, Agarwal G et al. Understanding why frequent users of EMS Call 9-1-1: a grounded theory study. Health Promot Pract [Internet]. 2018; Available from: http://journals.sagepub.com. Doi: 10.1177/1524839918799504 10.1177/1524839918799504 30222003

[14] Agarwal G, Lee J, Mcleod B, Mahmuda S, Howard M, Cockrell K (2019). Social factors in frequent callers: a description of isolation, poverty and quality of life in those calling emergency medical services frequently. BMC Public Health [Internet].

[15] Scott J, Strickland AP, Warner K, Dawson P. Describing and predicting frequent callers to an ambulance service: analysis of 1 year call data. Emerg Med J [Internet]. 2013;1–7. Available from: http://www.ncbi.nlm.nih.gov/pubmed/23413152 10.1136/emermed-2012-202146 23413152

[16] Leyenaar MS, Tavares W, Agarwal G, Costa AP (2019). Indicators of paramedic service use by community dwelling older adults. Ir J Paramed.

[17] Leyenaar MS, Strum R, Haque M, Nolan M, Sinha SK, Ontario Community Paramedicine Secretariat Steering Commitee. Report on the status of community paramedicine in Ontario [Internet]. 2019. Available from: https://www.ontariocpsecretariat.ca/resources

[18] BC Emergency Health Services. Community paramedicine in British Columbia improving health care in rural and remote communities [Internet]. 2017. Available from: http://www.bcehs.ca/our-services-site/Documents/CommunityParamedicine Initiative Overview.pdf

[19] Travers AH (2018). Evolution of a high-performance emergency health services system in Nova Scotia. Healthc Manag Forum.

[20] Alberta Health Services. 2019 Program performance report [Internet]. 2019. Available from: https://www.bja.gov/Publications/RSAT_PPR_Jan-Dec13.pdf

[21] Bigham BL, Kennedy SM, Drennan I, Morrison LJ (2013). Expanding paramedic scope of practice in the community: a systematic review of the literature. Prehospital Emerg Care.

[22] Gregg A, Tutek J, Leatherwood MD, Crawford W, Friend R, Crowther M et al. Systematic review of community paramedicine and EMS mobile integrated health care interventions in the United States. Popul Health Manag [Internet]. 2019; Available from: http://ovidsp.ovid.com/ovidweb.cgi?T=JS&PAGE=reference&D=medp&NEWS=N&AN=30614761 10.1089/pop.2018.0114 30614761

[23] Pang PS, Litzau M, Liao M, Herron J, Weinstein E, Weaver C (2019). Limited data to support improved outcomes after community paramedicine intervention: a systematic review. Am J Emerg Med [Internet].

[24] Thurman WA, Moczygemba LR, Tormey K, Hudzik A, Welton-Arndt L, Okoh C (2020). A scoping review of community paramedicine: evidence and implications for interprofessional practice. J Interprof Care [Internet].

[25] O’Meara P, Ruest M, Martin A. Integrating a community paramedicine program with local health, aged care and social services: an observational ethnographic study. Australas J Paramed [Internet]. 2015;12(5). Available from: http://ajp.paramedics.org/index.php/ajp/article/view/238

[26] O’Meara P, Stirling C, Ruest M. Community paramedicine model of care: an observational, ethnographic case study. BMC Health Serv Res [Internet]. 2016;16(1):39. Available from: http://www.biomedcentral.com/1472-6963/16/39 10.1186/s12913-016-1282-0 PMC4739332 26842850

[27] Jones A, Schumacher CR, Bronskill SE, Campitelli MA, Poss JW, Seow H, et al. The association between home care visits and same-day emergency department use: a case—crossover study. CMAJ [Internet]. 2018;190(17):E525–31. Available from: www.cmaj.ca/lookup/suppl/. Doi:10.1503/cmaj.170892/-/DC2%0Awww.cmaj.ca/lookup/doi/10.1503/cmaj.180263 10.1503/cmaj.170892 PMC5929891 29712671

[28] Verma AA, Klich J, Thurston A, Scantlebury J, Kiss A, Seddon G (2017). Paramedic-initiated home care referrals and use of home care and emergency medical services. Prehospital Emerg Care [Internet].

[29] Lee JS, Verbeek PR, Schull MJ, Calder L, Stiell IG, Trickett J, et al. Paramedics assessing Elders at Risk for Independence Loss (PERIL): Derivation, reliability and comparative effectiveness of a clinical prediction rule. CJEM [Internet]. 2016;18(02):121–32. Available from: http://www.journals.cambridge.org/abstract_S1481803516000142 10.1017/cem.2016.14 26988720

[30] Leyenaar MS, Strum RP, Batt AM, Sinha S, Nolan M, Agarwal G (2019). Examining consensus for a standardised patient assessment in community paramedicine home visits: a RAND/UCLA-modified Delphi Study. BMJ Open.

[31] CSA Group (2017). Community paramedicine: framework for program development.

[32] Morris JN, Fries BE, Bernabei R, Steel K, Ikegami N, Carpenter I et al. interRAI Home Care (HC) Assessment form and user’s manual. version 9. Washington, DC: interRAI; 2012. 5: 7–12

[33] Hogeveen SE, Chen J, Hirdes JP. Evaluation of data quality of interRAI assessments in home and community care. BMC Med Inform Decis Mak [Internet]. 2017;17(150):1–15. Available from: www.cihi.ca 10.1186/s12911-017-0547-9 PMC5663080 29084534

[34] Morris JN, Berg K, Björkgren M, Declercq A, Finne-Soveri H, Fries BE, et al. interRAI Community Health (CHA) Assessment form and user’s manual and related materials. version 9. Washington, DC: interRAI; 2010. 5, 6

[35] interRAI Canada [Internet]. University of Waterloo.2. 2020. Available from: https://uwaterloo.ca/interrai-canada/

[36] Heckman G, Gray L, Hirdes J (2013). Adressing health care needs for frail seniors in Canada: the role of interRAI instruments. Can Geriatr J.

[37] Leyenaar MS, McLeod B, Penhearow S, Strum R, Brydges M, Mercier E (2019). What do community paramedics assess? An environmental scan and content analysis of patient assessment in community paramedicine. CJEM Can J Emerg Med Care [Internet].

[38] Brohman M, Green M, Dixon J, Whittaker R, Fallon L. Community Paramedicine Remote Patient Monitoring (CPRPM): benefits evaluation and lessons learned [Internet]. Toronto, ON; 2018. Available from: https://infoway-inforoute.ca/en/what-we-do/news-events/webinars/resources/reports/benefits-evaluation/3542-community-paramedicine-remote-patient-monitoring-cprpm-benefits-evaluation-lessons-learned

[39] Martin-Misener R, Downe-Wamboldt B, Cain E, Girouard M (2009). Cost effectiveness and outcomes of a nurse practitioner–paramedic–family physician model of care: the Long and Brier Islands study. Prim Health Care Res Dev.

[40] Roggenkamp R, Andrew E, Nehme Z, Cox S, Smith K. Descriptive analysis of mental health-related presentations to emergency medical services. Prehospital Emerg Care [Internet]. 2018;22(4):1–7. Available from: http://ovidsp.ovid.com/ovidweb.cgi?T=JS&CSC=Y&NEWS=N&PAGE=fulltext&D=medp&AN=29364746%0Ahttp://oxfordsfx.hosted.exlibrisgroup.com/oxford?sid=OVID:medline&id=pmid:29364746&id=doi:10.1080%2F10903127.2017.1399181&issn=1090-3127&isbn=&volume=&issue=&spage=1&p 10.1080/10903127.2017.1399181 29364746

