# Supplementary material for: Paramedics assessing patients with complex comorbidities in community settings: results from the CARPE study
Source: CJEM. 2021 Aug 17;23(6):828–36. doi: 10.1007/s43678-021-00153-4 (PMC8575756; doi:10.1007/s43678-021-00153-4)
Supplement: Supplementary file 1 — Supplementary file1 (DOCX 16 kb) [file 43678_2021_153_MOESM1_ESM.docx]

SUPPLEMENTAL FIGURES

Figure 2 Mood symptoms and indicators of social supports of Community Paramedicine home visit patients, Home Care clients, and Community Support Services Agency clients. All mood symptoms as assessed over preceding 3 day period.

| * Evidence of symptoms of depression as determined by a score greater than or equal to 3 on the Depression Rating Scale. ** Modular assessment for Mental Health indicated within interCHA parameters. |
| --- |

Figure 3 Assessment of functional abilities and communication for Community Paramedicine home visit patients, Home Care clients, and Community Support Services clients.

| * Instrumental Activities of Daily Living where findings were not “Independent”. ** Activities of Daily Living where findings were not “Independent”. |
| --- |
