# Supplementary material for: Paramedics assessing patients with complex comorbidities in community settings: results from the CARPE study
Source: CJEM. 2021 Aug 17;23(6):828–36. doi: 10.1007/s43678-021-00153-4 (PMC8575756; doi:10.1007/s43678-021-00153-4)
Supplement: Supplementary file 2 — Supplementary file2 (DOCX 24 kb) [file 43678_2021_153_MOESM2_ESM.docx]

| Table S1: Identical assessment domains and items included in the interRAI HC, interRAI CHA, and Community Paramedicine assessment instruments | | | | | | |
| --- | --- | --- | --- | --- | --- | --- |
| Assessment Domains | DEMOGRAPHICS | LIVING ARRANGEMENT | ENVIRONMENTAL ASSESSMENT | | COGNITION | COMMUNI-CATION |
| Assessment items | Age  Gender | Lives Alone | *Home disrepair*  *Squalid conditions*  *Inadequate heating or cooling*  *Lack of personal safety*  *Limited access to rooms* | *Emergency assistance available*  *Access to grocery store*  *Home delivery of groceries available* | Daily decision making  Short-term memory | Making self-understood  Ability to understand others  Hearing  Vision |
| Assessment Domains | MOOD | | PSYCHOSOCIAL WELL-BEING | FUNCTIONAL STATUS | | |
| Assessment items | Negative statements  Anger  Unrealistic fears  Repetitive health complaints  Repetitive complaints (non-health related)  Sad or worried expressions  Crying or tearful  Withdrawal  Reduced social interactions | Self-report - less interest  Self-report - anxious  Self-report - sad | Social activities  Major life stressors  Time alone  *Family overwhelmed*  *Informal support* | IADL Capacity:  Meal preparation  Housework  Managing finances  Managing medications  Phone use  Stairs  Shopping  Transportation | ADL Self-performance: Bathing  Personal hygiene  Dressing upper body  Dressing lower body  Walking  Locomotion  *Transfer toilet*  *Toilet use*  *Bed mobility*  *Eating* | Mode of locomotion  Days went out |
| Assessment Domains | CONTINENCE | DISEASE DIAGNOSES | | HEALTH CONDITIONS | | |
| Assessment items | Bladder continence  *Bowels continence* | Alzheimers  Dementia  Stroke/CVA  Cardiac disease  COPD  CHF | Anxiety  Depression  Schizophrenia *Pneumonia*  *UTI (past 30 days)*  Cancer  Diabetes  Multiple Chronic Diseases | Dizziness  Unsteady gait  Chest pain  Abnormal thoughts  Delusions  Hallucinations | Acid reflux  Constipation  Diarrhea  Vomiting  Unable to sleep  Sleeping more than normal  *Peripheral edema*  *Aphasia* | Falls (past 30 days)  Dyspnea  Fatigue  Unstable health conditions  Self-reported health  Tobacco use |
| Assessment Domains | NUTRITION | MEDICATIONS | TREATMENTS | |  |  |
| Assessment items | Weight loss  Decreased food/fluid intake | Drug adherence | Dialysis  Oxygen therapy  Transfusions  Wound care | Palliative care Overnight hospital stay  ED visit | *Italics* indicate assessment items available as part of the interRAI CHA Functional Supplement (not included in reported analyses) | |
